# The Effects of the Opioid Crisis on Agricultural Industries

**DOI:** 10.3390/ijerph19095343

**Published:** 2022-04-27

**Authors:** Heidi Liss Radunovich, Terasa Younker, Jillian M. Rung, Meredith S. Berry

**Affiliations:** 1 Department of Family, Youth and Community Sciences, Institute of Food and Agricultural Sciences, University of Florida, Gainesville, FL 32611, USA; tyounker@ufl.edu; 2 Department of Health Education and Behavior, College of Health and Human Performance, University of Florida, Gainesville, FL 32611, USA; jrung@ufl.edu (J.M.R.); mberry@ufl.edu (M.S.B.); 3 Department of Psychology, College of Liberal Arts and Sciences, University of Florida, Gainesville, FL 32611, USA

**Keywords:** opioid use, agriculture, mental health, depression, pain, stress

## Abstract

Opioid use remains a significant public health crisis. However, few quantitative or qualitative data exist on the prevalence of opioid use and associated mental health conditions in agricultural industries and how it affects the industries themselves. Data on opioid use and associated consequences were collected among agricultural business owners and workers using both quantitative (*n* = 129) and qualitative assessment (*n* = 7). The prevalence of opioid use, pain, stress, and depressive symptoms as well as associated hazards were characterized among individuals who work in horticulture (nursery and landscape) and those who work in food production (livestock and crops). Qualitative interviews were also conducted to better understand individual experiences with opioid use. Opioid use was significantly higher among horticultural industries compared to food production. Pain and depressive scores were higher among those who had used opioids although stress did not differ. Importantly, substantial percentages of participants who reported opioid use also reported consequences associated with their use, including missing work, being injured at work while using, and having difficulty in completing daily tasks. These results provide initial evidence that opioid use is substantially affecting agricultural industries in terms of mental health, personal health, labor availability, and productivity.

## 1. Introduction

The rising incidence of opioid use and its associated aftermath have been deemed a public health crisis in the U.S. [1,2]. The term opioid encompasses prescription opioid medication (used to treat pain, most commonly oxycodone and hydrocodone), the synthetic opioid fentanyl (used for severe pain), and heroin (an illegal opioid in the U.S.) [3]. Although opioids can be effective for treating some painful conditions in the short term, some individuals may develop harmful opioid use or opioid use disorder.

The number of opioid prescriptions to treat pain in the U.S. rose tremendously between 1999 and 2015, increasing by nearly four-fold, which seemed to fuel the initial stages of the opioid crisis [1]. This was worrisome because the more prescriptions provided, the greater the likelihood of addiction and use of prescribed medicine by others, and those who are prescribed opioid medications are at greater risk for later using the illegal opioid heroin [1,3]. After efforts to educate physicians about the dangers of prescribing opioids and legislation to limit their use, recent statistics suggest a shift in the number of prescriptions written although overall opioid use and overdose have continued to increase [4]. It is notable that there are additional pathways to potential harmful opioid use in which the benefits and potential harms must be weighed that may or may not include initial prescription use (e.g., managing emotional pain) [3].

While the numbers of prescriptions for opioids have decreased recently, there has continued to be an alarming increase in opioid-related deaths in recent years with the rise of the use of fentanyl, and the COVID-19 pandemic also has been associated with the rise in rates [4]. According to the National Institute of Drug Abuse (NIDA), in 2020, the majority of drug overdose deaths in the U.S. (nearly 75%) were related to opioid use, accounting for over 68,000 deaths [5]. The opioid-associated number of deaths increased by more than five-fold between 2015 and 2020 [5]. Non-fatal opioid use also imposes substantial financial burden and significant consequences to individuals as well as organizations. Increases in absenteeism, slower reaction time, less productivity, more safety hazards, and higher costs for insurance are all common when there is use of opioids by personnel [6]. There are also additional societal costs, such as increased criminality and burden to the penal system, greater strain on families as evidenced by greater need for foster care, and developmental impacts on babies born addicted to opiates [7].

### Impact on Agricultural Industries

Given the potentially destructive nature of opioids and the importance of agricultural industries to the well-being and security of U.S. residents, it is important to gain a better understanding of the impact of harmful opioid use on agricultural industries as well as areas of need for resource and program development. Levels of opioid use and associated consequences vary tremendously by location even within a given state. Indeed, opioid use and associated issues tend to vary by urban and rural areas, with differing risk factors, consequences, and resources [8]. In a survey conducted for the American Farm Bureau Federation, around 74% of those in agricultural industries reported being directly impacted by opioid use [9]. Environmental stressors, such as sun exposure, heavy lifting, long hours, weather fluctuations, and financial stress, are present to place those who work in agricultural industries in danger of harmful opioid use; the risk of injury and need for pain relief is greater while working in agricultural sectors than in many other occupations, and opioid use deaths for those in the farming, fishing, and forestry industries are more than five times higher than for the general population of workers [10,11].

While agricultural industries seem to have disproportionate experience with harmful opioid use, what remains unclear is how the prevalence of opioid use is affecting the industries themselves. Opioid use has the potential to reduce the available workforce, increase safety hazards at the worksite, increase worker turnover, and increase health issues for workers and their families. However, the extent of this impact has not yet been assessed. It is important to understand not only the extent of the problem but how specific agricultural industries might be differentially impacted. Furthermore, understanding the correlates of opioid use in agricultural industries as well as its impact on the industries and population is necessary. Recent research suggests that farmers are experiencing high rates of stress and depression; however, pain also appears to be strongly associated with harmful opioid use [12]. A further understanding of how these issues relate to opioid use among agricultural industries can help inform avenues for preventive efforts.

This study provided a quantitative and qualitative examination of how opioids broadly impact agricultural industries and their communities within a single U.S. state. Specifically, this study was designed to understand: the prevalence of opioid use and how it affects those within agricultural communities; associations between opioid use and potential contributing factors such as depression, stress, and pain; and how these contributing factors may differ across sectors of agricultural industries.

To achieve these goals, community members working in agricultural industries in a single state were recruited to participate in either a quantitative survey and/or a structured, open-ended interview. In the former, we asked individuals about the type of agricultural industry they worked in, the different ways in which the opioid use has potentially impacted their workplace (e.g., worker turnover, changes in productivity, absences), personal opioid use and that of close others, the impact of opioids on one’s own work, and included standard measures of depressive symptoms, stress, and pain. In qualitative interviews, participants’ awareness of the opioid epidemic, opioid use, their unique experiences with opioid use (theirs, family members, etc.), and thoughts about actions that may help were elicited. Together, these quantitative and qualitative data help produce an understanding of the scope of the opioid epidemic in agricultural industries, differences in the way it may present, and new avenues to reduce its impact.

## 2. Materials and Methods

### 2.1. Participants

Both studies (quantitative and qualitative) were approved by the university’s Institutional Review Board. All participants were recruited via advertisements through partnering organizations, such as the Farm Bureau, the (blinded sponsoring center), and specialty associations through their member channels, including e-mail announcements, Facebook posts, and newsletters. Individuals interested in participating in the quantitative study accessed the study via link, and those interested in participating in the interview were directed to contact the principal investigator to learn more about the study and/or to schedule an interview. Multiple attempts at recruitment were made over a series of months, and recruitment ended when no additional participants joined the study after a period of one month of active recruiting, suggesting that there were no additional participants willing to complete study procedures.

Participants in the quantitative study could obtain a USD 10 store gift card for completing the survey but needed to provide contact information in the survey in order to receive the gift card. Many participants did not provide the needed information to receive the gift card (name and address). Individuals participating in the qualitative study were eligible to receive a USD 25 gift card as compensation for participation; one participant declined to receive compensation.

### 2.2. Procedures

Data collection for the quantitative portion of the study took place from March to August 2019, within the year before the start of the COVID-19 pandemic. Interested individuals provided informed consent online in Qualtrics, and those who consented were then asked questions to gauge their eligibility. These questions pertained to location (whether individuals lived in the state) and occupation (whether individuals worked in one of the agricultural industries of interest). For the latter, the three industries of interest were livestock, crops, and nursery/landscape. If individuals did not qualify, the survey was terminated. Those who did qualify proceeded to the remainder of the survey, which contained the measures outlined in the respective section below. At the end of the survey, individuals were asked to provide their contact information so that study staff could send their compensation if desired.

Qualitative data collection took place from March to September, 2020—early in the COVID-19 pandemic. Potential participants reviewed the study procedures with the lead researcher via phone and provided consent electronically via DocuSign. After consent, the participants were referred to the (blinded) Center to schedule an interview. Interviews were conducted by trained (blinded) Center staff members. Participants completed the interview via Zoom, which took between 10 and 30 min to complete. Interviews were structured, with some questions specific to business owners. Interviews were recorded for subsequent transcription. After transcription and de-identification, the original recordings were destroyed. Analysis was conducted by a research associate trained in qualitative analysis.

### 2.3. Quantitative Study Measures

In order to quantitatively examine opioid impact on agricultural industries, the following measures were utilized. All measures were completed in a survey using Qualtrics and appeared in the order presented below.

#### 2.3.1. Investigator-Generated Demographic and Opioid-Related Questionnaire

This measure included demographic items of interest related to industry, age range, and whether or not the participant was an owner of an agricultural business. Additional questions tapped into opioid use, knowledge of others who use opioids, impact on the business, and perceptions of impact on family. Items were generated by the researchers, reviewed by several volunteers, and revised for clarity. The questionnaire consisted of 22 items presented to all participants, with skip logic used to only present opioid use questions to those who identified as using opioids. An additional 8 questions pertaining to business and employee impact were only presented to individuals identifying as business owners or co-owners.

#### 2.3.2. Perceived Stress Scale (PSS)

The PSS is a brief, ten-item instrument that is widely used to assess the degree to which people feel their life is stressful [13]. The ten-item version is adapted from an initial 14-item version [14]. Each item of the PSS is a statement pertaining to the frequency of feeling different types of stress or feelings related to stress in the past month. An example statement from the PSS is, “In the last month, how often have you felt that you were unable to control the important things in your life?” Responses are provided on a scale of 0 (never) to 4 (very often), and the scale score is the sum of the individual item scores. The scale has good internal consistency (alpha = 0.86) and convergent validity (e.g., scores significantly correlate with conditions associated with stress such as depression and anxiety) [15]. The PSS also has good test-retest reliability, with studies reporting correlations of 0.70 or greater [16].

#### 2.3.3. Center for Epidemiologic Studies Depression Scale, Revised (CESD-R)

The CESD is a measure of depressive symptomatology that was designed for community samples, whose items were developed from those present in other validated depression questionnaires [17]. It was later revised in order to better align with DSM diagnostic criteria for a depressive episode [18]. The CESD-R consists of 20 statements that reflect feelings over the past week that positively or negatively correlate with depression (e.g., “I was bothered by things that usually don’t bother me” versus “I was happy”). Statements are rated based on the frequency the participant felt the stated way during the past week, with four frequencies ranging from “Rarely or none of the time (less than 1 day)” to “Most or all of the time (5–7 days)”. Low frequencies receive a score of 0 and high frequencies a score of 4. Positive items are reverse-scored. The total score for the CESD-R is the sum of scores across the 20 items. The CESD-R has good internal consistency (alpha > 0.92) and correlates well with other depression measures [19].

#### 2.3.4. McGill Pain Questionnaire—Short Form

The Short Form of the McGill Pain Questionnaire (MPQ) [20] elicits current pain levels according to 11 different sensory dimensions (e.g., cramping, gnawing), different types of affective impact (and intensity thereof; e.g., sickening, fearful), and overall intensity of pain experienced. There are three primary scores from the questionnaire: the sum of the pain level ratings across different sensory qualities, the sum of the affective intensities, and an overall score summing all sensory and affective ratings. Each sensory and affective item is rated on a four-point scale ranging from none (0) to severe (3). Scores significantly correlate within individuals prior to and after receiving an analgesic treatment for pain; and scores decrease significantly following analgesic treatment [20].

### 2.4. Quantitative Data Analyses

Because the majority of study aims were descriptive in nature, the survey results are primarily discussed as such. Most survey questions were closed-ended, and responses were nominal or ordinal in nature; thus, frequencies and percentages are used to discuss the majority of findings. For evaluating whether there were differences in the presence and impact of the opioid epidemic by industry (nursery and landscaping vs. livestock and crops), the distribution of responses to 10 key questions were compared using Fisher’s Exact tests. The key questions pertained to: knowledge and experience with the opioid epidemic (familiarity with, knowing individuals who use opioids, and personal opioid use); the ways in which opioids have affected businesses (specific types of impact, worker turnover); and the prevalence of opioid-related problems in the workplace (proportion of workforce impacted by opioids, injury at work while using opioids, quitting or getting fired due to opioid use). Finally, scores of depressive symptoms (CESD-R), stress (PSS), and pain (MPQ) questionnaires were compared across industry groups using Wilcoxon signed-rank tests. The distributions of the aforementioned questionnaire scores evidenced non-normality, as determined by Shapiro–Wilk tests; thus, the non-parametric counterpart to the *t*-test was chosen for these analyses.

### 2.5. Qualitative Interview Content Analysis

Thematic content analysis on transcribed interviews was conducted with the aid of coding software NVivo 10 (QSR International, Burlington, MA, USA). The analyst began by reading and re-reading each interview to gain a general idea of their content, taking notes both in the margins of the uploaded interview documents and on a separate document. Code words and phrases were noted to accurately describe a text segment: for example, the term “family troubles” was used to indicate transcript sections referring to family problems caused by or related to opioid dependence. These codes were then re-examined and condensed into larger categories. Major themes were identified by the frequency of coded sections and their relationship to larger forces or structures.

## 3. Results

### 3.1. Quantitative Findings

After data cleaning, a final sample of 129 individuals participated in the quantitative survey and were included in the analytic sample. A majority of participants identified as working in horticultural industries (*n* = 80; 62.0%), and many identified themselves as owners or co-owners of an agricultural business (*n* = 74, 57.4%) with a predominantly (52.7%) moderately-sized workforce (21–40 full- and part-time workers). Many participants were younger to middle-aged adults in the age ranges of 26–35 years (*n* = 43, 33.3%) and 36–45 years (*n* = 61, 47.3%). Table 1 provides further details on participants’ ages for the full sample and by industry type.

#### 3.1.1. Opioid Knowledge and Experience in the Full Sample

In the full sample, participants were generally knowledgeable about the opioid epidemic. The majority of individuals (59.7%) reported having at least “heard quite a few things” about the opioid epidemic, with it having varying degrees of personal impact. Among those that had heard quite a few things or more (*n* = 77), 48.1% reported the opioid epidemic having at least some impact on them. It was more common that these participants reported the opioid epidemic having some impact (39.0%) as opposed to a significant impact on their life (9.1%).

Many participants reported knowing someone who used opioids (*n* = 89, 69.0%) and personally using opioids at some point in their lives (*n* = 70, 54.3%). Most individuals who knew someone else who used opioids reported that a family member had used them (*n* = 72, 80.9%). Of those that had used opioids, most of participants’ use resulted from a prescription provided for a work-related injury (77.1%). At the time of the prescription, participants were often provided some information about use of prescription opioids, but many reported still having questions about use (50.0%). Others indicated receiving ample or adequate information (44.3%), with very few indicating that opioid use was not discussed (5.7%).

Even though opioid use at least began as and may have continued to primarily constitute medical use, many participants reported feelings of guilt or shame with their opioid use (52.9%). Many of these participants who had used opioids reported receiving a diagnosis of opioid use disorder by a licensed professional (*n* = 46, 65.7%). Of those who reported being diagnosed with opioid use disorder, the majority reported seeking treatment for their usage (80.4%).

#### 3.1.2. Work and Personal Impacts of Opioid Use

Participants were well-informed about drug policies in the workplace, with few participants indicating uncertainty about such policies existing (4.7%). Of those aware of their employer’s status of having a drug policy (*n* = 123), it was more typical than not to have one (81.3%). Those who had used opioids (*n* = 70) reported that they sustained injuries at work at fairly high rates (68.6%), that opioids frequently interfered with their ability to complete daily tasks (64.3%), and had at some point resulted in absence from work (54.3%). Some participants reported quitting or being fired from a job due to their opioid use (47.1%).

In the same subset of participants, a variety of general health impacts of opioid use were reported. Many individuals reported experiencing withdrawal symptoms from opioid use (58.6%), insomnia (68.6%), and anxiety (67.1%). Confirmation of other specific health effects were less frequent, albeit still relatively common (e.g., memory problems, 42.9%; mood swings, 31.4%), and few participants indicated that their health was not at all impacted by opioid use (7.1%).

#### 3.1.3. Owner and Co-Owner-Reported Opioid Impacts on Work

A very large percentage (94.6%) of the owners and co-owners of agricultural businesses surveyed (*n* = 74) reported having at least one employee who had problems related to opioid use. The most common response was having four or more employees who had problems with opioid use (*n* = 26, 35.1%). Translating these employee numbers to proportion of workforce, most owners and co-owners reported that more than none but less than half of their workforce is believed to be affected by opioids (*n* = 45, 60.8%). Owners and co-owners also most frequently indicated that four or more employees have had an opioid overdose (*n* = 28, 37.8%); the majority of owners and co-owners reported having at least one employee who overdosed (*n* = 63, 85.1%).

When asked about the primary way opioids have impacted participants’ businesses, two were especially prevalent. The most frequent was a decrease in employees, which was indicated by 36.5% of owners and co-owners. A potential contributor to the reduced number of employees is greater turnover, which was reported as an issue by 55.4% of business owners/co-owners (across additional response options of “No” and “Unsure”). The second most frequently endorsed impact of opioid use on businesses was a decrease in productivity (29.7%), which all owners and co-owners estimated to be by a magnitude of 20% (70.3% of owners and co-owners; relative to options of not reducing productivity, reducing productivity by 50%, or being unsure). Other impacts (e.g., coming to work high) were generally endorsed at similar, lower frequencies (between 8.1 and 12.2%) although most participants reported that opioid use was associated with absences for varying reasons (*n* = 65, 87.8%)—most commonly, for substance use treatment (55.4%).

#### 3.1.4. Differences across Industries

Due to the relatively lower numbers of participants in crop and livestock industries, a decision was made to limit comparisons to those in production agriculture (crops and livestock) versus those in horticultural agriculture (nursery and landscape) rather than considering each industry separately. Overall, those in horticultural industries appeared to be more heavily impacted or differentially impacted by the opioid epidemic than those working in production industries. Of the 10 questions targeted for group comparisons, all but one showed significant industry differences, which was the frequency of experiencing injury at work while under the influence of opioids. The frequencies and percentages of individuals selecting each response option for the 10 target questions, along with the corresponding *p*-value from Fisher’s exact tests, are shown in Table 2.

Among individuals of the different industries, there were similar levels of awareness of the opioid epidemic, but those in production industries reported being personally impacted more frequently. Despite higher endorsement of being personally impacted by the opioid epidemic, those in production industries were less likely to report knowing someone who uses opioids (49.0%) and to have used opioids themselves (24.5%) than those in nursery and landscaping (with 81.3% and 72.5% knowing someone who used opioids and reported personally using opioids, respectively). Lower rates of opioid use in production industries coincided with lower frequencies of reported clinician-diagnosed opioid use disorder (30.8%) than in horticultural industries (72.4%) as well as lower incidences of quitting or being fired for opioid use (23.1% vs. 51.7%).

Owners and co-owners of horticultural businesses reported greater proportions of their workforce being impacted by opioid use and were more likely to experience worker turnover as a result of opioid use (62.3% vs. 38.1%). Those owning production agriculture businesses were more likely to report a decrease in employees as the primary impact of opioid use (42.86%), whereas those owning production businesses noted that a decrease in employees (35.85%) and decrease in productivity (33.9%) were the primary issues reported at relatively similar frequencies.

Factors previously shown to be associated with opioid use (depression, stress, and pain) also revealed industry-related differences. Those working in horticultural industries experienced greater levels of depressive symptoms (*Mdn* = 30, Q_1_ = 14, Q_3_ = 33; *n* = 71) than those in production industries (*Mdn* = 19, Q_1_ = 12, Q_3_ = 25; *n* = 45), which was supported by significantly higher CESD-R scores (*W* = 1119.5, *p* = 0.007). The prevalence of experiencing pain in the total sample was high (71.1%) and more so among those in horticulture (81.1% vs. 55.1%). Those in horticultural industries also reported experiencing more severe pain (*Mdn* = 20, Q_1_ = 7, Q_3_ = 24; *n* = 65) on the MPQ than those in production industries (*Mdn* = 6, Q_1_ = 3, Q_3_ = 12; *n* = 26), *W* = 455, *p* < 0.001. There were no significant differences between industry groups in perceived stress (*p* = 0.10); however, it is notable that a high level of stress was reported across participants, which may have led to a ceiling effect.

### 3.2. Qualitative Findings

All seven respondents in the qualitative sub-study were agricultural owners/producers rather than workers. Four were male, and ages ranged from 28 to 63, with an average age of 51. Four respondents worked in the livestock industry, two in the crop industry, one in dairy, one in forestry, and one in horticulture. Two older male respondents were current physician-prescribed opioid users. Data analysis resulted in the emergence of five distinct but interrelated themes: (a) awareness of the epidemic, (b) effects of opioids, (c) agricultural injury and chronic pain, (f) recommended interventions, and (g) heartbreak and compassion.

#### 3.2.1. Awareness of the Epidemic

All seven respondents reported strong awareness of the scale and severity of the opioid epidemic. All were either opioid users themselves or knew someone who used opioids. Four respondents reported having family members, two reported having friends or acquaintances, and two reported having employees who were addicted to opioids. There was a strong recognition of the need for a more robust response to the crisis, with three respondents explicitly asking for additional ways to aid in the study in order to develop more effective interventions.

##### Over-Prescription

All respondents made some reference to the problem of over-prescription of opioids and six to addiction problems originating from legal prescriptions for pain-management. A participant stated, “It’s doctors because people go to a doctor, and they think they know, and they have their best interest, but they just prescribe these things...you don’t know what that prescription’s gonna do to you for the rest of your life.” Four respondents discussed opioid addiction within the context of a greater problem of drug use and addiction: “Whether it’s the opioid epidemic, the benzo epidemic, but it’s just, for me, I see the overarching theme is the dependency because of the—just the shelling out prescriptions.”

##### Lack of Adequate Risk Education by Medical Professionals

The two respondents taking opioids for pain management both reported being provided with inadequate information on the dangers of opioids before receiving prescriptions. A participant who was prescribed fentanyl after a botched shoulder surgery blamed inadequate addiction education on his decision to start fentanyl in the first place: “I wish when we first started this, I would say I wasn’t as aware of what was involved with the addiction… had I understood at the time how difficult it would be to eliminate the fentanyl, I would’ve told the doctor no, I don’t want the fentanyl. I’ll just stay with the pills.”

#### 3.2.2. Effects of Opioids

All respondents described the effects of opioids and opioid use on both users and non-users in striking detail.

##### Effects on Users

Those who used opioids themselves were well-aware of the addictive nature of their medications as well as the physiological effects (drowsiness, forgetfulness). As expected, opioid use increased safety hazards at the worksite. A respondent taking opioids as prescribed for pain management frankly admitted the negative impact of opioids on his ability to perform his farmwork safely: “You do stupid things sometimes. You really do. When you’re running equipment or whatever, that can be very, very dangerous. I’ve learned to just be very, very cautious, and write things down, and go through procedures in my head before I do them. Yeah, opioids and machinery are a scary thing.”

Producers with employees reported firing employees they suspected of using opioids rather than risk them injuring themselves or others at work: “Depending on what their responsibilities are, termination may be the only choice. You can’t have someone on opioids on heavy equipment, for example…There’s lots of things they can’t do, dangerous, hazardous work, which most of what we do is dangerous and hazardous in some form.” Furthermore, in some cases, this participant’s comment holds true for use in line with prescriptions. Individuals who did not use opioids reported witnessing first-hand and suffered the consequences of behavioral (lying, stealing, decreased job performance), psychological (emotional distress, personality change, psychosis), and physiological symptoms (exhaustion, infection from needle sharing) of dependence on opioids among those close to them.

##### Effects on Individuals Who Do Not Use Opioids

The individuals who did not report opioid use also reported psychological, monetary, legal, emotional, and moral collateral damage from the opioid epidemic. Those whose family members engaged in opioid use reported high levels of family strife and division, leading to individual psychological trauma. One participant whose son had died the previous year from a contaminated opioid needle reported ongoing spousal conflict and “mental pain” even a year after her son’s death. A participant with multiple family members who have substance use disorders expressed the heartache of unambiguous loss and drug addiction: “I can just say it’s a heartbreaker. You have to mourn the loss of somebody without actually losing them. That’s something to come to terms with.” Producers trying to support family members who engaged in harmful substance use also reported endlessly sacrificing time, money, and attention to help their loved one: “At home I’ve been affected financially, emotionally, very long term. We’ve gone through everything from dealing with the first arrest, car wrecks, five vehicles over a 10-year period, three arrests, lawyers, court dates, rehab three times. You name it, we’ve probably have been through it and are continuing to deal with it.”

##### Effects on Businesses

Four of the seven respondents reported the epidemic as affecting their businesses. Three respondents reported widespread addiction among the agricultural workforce and difficulty finding workers who were not addicted to opioids: “Many of the young men that we’ve hired over the years are from rural (blinded county). Apparently, there is quite an opioid problem out in that area because most, if not all, the young men we’ve hired from out there have come in with addiction issues,” reported a crop and cattle owner. “When it comes to hiring workers, basically, as long as they’re not a meth or heroin addict, it’s like, okay, great. It’s so hard to find [drug-negative workers] in that area: fentanyl, oxy, hydromorphone, heroin, methadone, oxycodone, percocet, hydrocodone, morphine.”

Two respondents described how their businesses have suffered due to increased liability costs, decreased productivity, high levels of conflict, and dishonesty among workers who engage in harmful drug use.

“As a business, your insurance goes up when you have more accidents. If your employees are found to be under the influence that definitely affects the cost of purchasing insurance in the future. Productivity absolutely drops off. After an addict is employed for a few weeks and they get comfortable, they tend to exit before the week is over, midday on Friday, and show up late on Monday. As they get more comfortable, if they’re not fired immediately, they become more intolerant and leave earlier, stay gone longer and create a lack of productivity in whatever crew they’re in. Eventually they get fired. It’s a vicious cycle.”

Not having employees did not necessarily protect employers from direct business losses. One respondent reported her opioid-dependent son stealing USD 10,000 in farm equipment in order to buy drugs.

#### 3.2.3. Agricultural Injury and Chronic Pain

Five of the seven respondents explicitly linked the hazards of farmwork and chronic pain issues in the agricultural population to high risk for opioid dependence. Three respondents reported experiencing chronic pain. All three described pain as an inescapable part of agricultural life and the strong mental fortitude necessary to keep working. “I will say to you that I know of people that are in the farming business, and in the farming business we’re always hurting our hands or legs. Anytime we’re messing with heavy equipment or dealing with animals we’re gonna get mashed, pushed around, so we live with pain or deal with pain. It’s just part of what we do.”

Two respondents used opioids to manage the pain, but one had recently cut his usage in half, and the other reported his intention to stop completely. One individual reported receiving acupuncture treatments and taking muscle relaxants in addition to his prescribed opioids and that he had tried other pain-management medications and treatments to little effect. An individual who did not use opioids reported engaging in recreation (“sports, golf, fishing, stuff like that”) to manage his pain.

#### 3.2.4. Recommended Interventions

When asked what they thought would help with the opioid epidemic, responses fell squarely into two categories: better opioid education and tighter regulations on prescriptions.

##### Education

There was wide consensus among the respondents regarding the need for improved education about opioids. Four reported being unaware of any beneficial educational resources. Both opioid users expressed dismay at how little information they had received about the addictive nature of opioids before receiving prescriptions, and two respondents expressed frustration at the difficulty of accessing educational resources: “I think that the education resources are very difficult to locate. I’m not so sure that they don’t exist, but they’re not readily available to folks that are in need.” “I think there needs to be more resources out there on the streets for people to know where they can get help. I know when I was going through this, I had to learn it all on my own.”

Two respondents proposed school-based opioid education programs for young people: “[We need] to really try to imprint it on our youth in the schools exactly the cost that doing those types of drugs has cost us as a society,” explained a participant. “We’re putting our youth out into the world and they don’t know life’s basics. This [harmful opioid use] is sadly becoming a life basic, it really is.” A participant who had been an educator for 30 years also saw value in school-based programs for young people: “it’s getting them early, and educating them, and making sure they understand these things can ruin your life.”

##### Restrictions on Prescription Opioids

Four respondents explicitly blamed over-prescription as the primary cause of the epidemic and endorsed much stricter prescribing regulations, with one respondent going as far as proposing to “shut down the pharmacies.” Three respondents mentioned the effectiveness of recently tightened regulations on pharmacies: “I think there’s a lot more regulation in the pharmacy industry where people who doctor shop and things like that. Maybe even doctors. I know there was one in a neighboring town that was arrested for over-prescribing, a dentist. I think that’s good. Hopefully some doctors are being scared into not doing that.”

#### 3.2.5. Heartache and Compassion

One encouraging theme that emerged in these interviews was the heartbreak and compassion expressed toward those struggling with opioid addiction. Perhaps because doctors and pharmacies were perceived as the perpetrators of the crisis, expressions of understanding, compassion, and pathos for the suffering far outweighed those of frustration and anger. “It is a very telling fact that most of the addicts in our industry are probably…addicted from an initial doctor’s office visit with a prescription. It’s not from going out there partying and things like that. May become that. May become something that they have to do physically to get through the day, but it starts out with I sprained my back or I twisted my knee, and the doctor gives you something. Then you take it. Some folks can stop taking it…some other folks are out on the street corner buying it for the rest of their lives.”

When asked how they would respond to an employee with an opioid problem, all but one respondent said they would do everything they could to get said employee into treatment: “Personally, we would try to help them in any way. We would send them to rehab. We would send them anywhere to get them help for X amount of time.” Two respondents reported volunteering in initiatives battling the epidemic (needle exchange and an employee assistance program). Ironically, however, this compassion and desire to help individuals who use opioids often ended up being costly—both financially and psychologically. One participant reported taking multiple employees to rehab and expressed his frustration simply: “We’re here to run a business, we’re not social workers.”

## 4. Discussion

Little research exists characterizing opioid use among agricultural populations, and to our knowledge, this is the first study engaging in both quantitative and qualitative examination of opioid use among agricultural populations in the United States. Despite the relatively small sample size, it elucidates the concrete ways in which agricultural workers and producers are affected by and are responding to the opioid crisis. As such, it provides much needed research in an under-researched topic area and a foundation for further research.

Participants’ quantitative survey responses revealed that the opioid epidemic is generally well-recognized in agricultural industries, with nearly half of the sample having been impacted by it to varying degrees. The estimate of those impacted in the present study was similar to that previously published by the American Farm Bureau Federation [9] specifically among those in food production industries (crops and livestock); however, our study suggests that those in horticultural industries (nursery and landscaping) show higher self-reported rates of opioid use and dependence.

Previous research has suggested that there are environmental stressors associated with work in agricultural industries that increase their vulnerability to opioid use, and those who work in agricultural fields have shown a greater than five-fold higher incidence of opioid overdose deaths [10,11]. In the current study, the nature of work in agricultural industries disproportionately exposing individuals to opioid dependence was supported. More than half of the total sample had used opioids—which was by and large due to work-related injury—and more than half of those who had used opioids developed dependence. Opioid use also substantively exposed individuals to additional risks, including economic (quitting or being fired from one’s job) and physical (injury), with the latter receiving parallel support from interview data. The risk of opioid use and dependence was especially high among those in horticultural industries, who also had elevated levels of depression and experiences of pain. It may be that greater experience of pain leads to opioid use; however, hyperalgesia (increased pain sensitivity) is a commonly experienced side effect of continued opioid use. Considering this and that the frequency of prescriptions for opioid use as a function of work-related injury was not significantly different across industry types, it is possible that the elevated pain (and perhaps depressive symptoms) in this sample could be an effect of opioid use itself. However, due to the lack of additional temporal information, the specific cause of heightened pain and depression in this group remains uncertain. It is also possible that industry-specific types of work might lead to higher rates of pain. For example, nursery and landscaping, which involves the growing, digging, transporting, and replanting of trees, shrubbery, turfgrass, or other plants, may require more direct physical labor, whereas heavy-duty labor might be more likely to be mechanized in crop industries, and both may vary from animal-oriented livestock production.

Interviews often corroborated the quantitative survey data but provided additional insights. The information yielded from interviews further emphasized the impact of opioid addiction in increased health issues for workers and their families. Even owners with no employees using opioids saw their businesses affected by personal and family trauma due to opioid use.

Overall, these data showed that harmful opioid use resulted in a reduced available workforce, increased worker turnover, and financial toll from the perspective of business owners. In particular, owners and co-owners of horticultural businesses reported greater proportions of their workforce being impacted by opioid use and were more likely to experience worker turnover as a result of opioid use. The horticultural industries, therefore, might be a particular population on which to focus future resources and focused interventions.

### 4.1. Implications for Practice

This research has the potential to benefit policy makers and practitioners regarding how to develop effective and tailored interventions for this population. First and foremost, this research demonstrated the urgent need for increased dissemination of opioid-related resources within the agricultural population. The awareness, compassion, and willingness of the participants in this sample to help those affected suggests agricultural owners to be invaluable human resources in stigma-reduction and other education campaigns. The fact that more than half of these respondents reported being unaware of helpful opioid-related resources is an unfortunate and relatively easily remedied situation. Considering agricultural owners’ awareness of the issue and the diverse ways in which they are impacted, they should be considered key stakeholders and included in discussions of community-based solutions.

Relatively high rates of pain, stress, and depression were observed in this population, and these associated conditions may represent a target for intervening prior to developing harmful opioid use or to supplement ongoing treatment of opioid use. Developing accessible, affordable, and widely distributed non-opioid pain mitigation strategies (e.g., readily available virtual support for physical pain from healthcare professionals such as physical therapists) could facilitate both education and recovery from injury without opioids. Psychological support that is also accessible and affordable in rural areas (e.g., virtual therapist meetings) could also facilitate the treatment of co-occurring mental health conditions to enable healthy lifestyle changes. Access to pharmacotherapy (e.g., methadone, buprenorphine, naltrexone), which is the standard of care for opioid use disorder and opioid use, can also be difficult to obtain in some rural areas due to long distances from treatment centers and required daily dosing. Increasing such access (e.g., more rural methadone clinics as well as access to buprenorphine and naltrexone) could have major benefits to the community in reducing the individual, psychological, industry, and financial cost of the opioid crisis particularly in rural areas and among agricultural industries. Finally, proactive engagement in preventative education regarding opioid use as well as safety-related training and kinesthetic changes to work routines can help reduce the likelihood of opioid use.

Third, this research demonstrates the need for increased support—monetary, legal, emotional/psychological, moral—for those in affected communities who do not use opioids. Employers struggling with worker use of opioids suffer monetary, legal, and moral setbacks and dilemmas that affect both their bottom line and their own psychological wellbeing. Recommended interventions, such as financial support, legal aid, and mental health services, need to be considered for affected non-users.

### 4.2. Limitations

There are several limitations to this research. One limitation of the study is its sample size, which did not allow for separate analysis by gender, age, or industry. Further, all participants were recruited from a single state within the U.S., and it is possible that impact and associated issues may vary from state to state or by country, so it is recommended that future research expand the scope to include broader samples. Due to the non-random online recruitment process results, participants with a strong interest in the opioid epidemic could be overrepresented in the sample. Both qualitative and quantitative studies relied on self-report, and participants may not have been entirely open when speaking about highly stigmatized behaviors such as drug use. Some survey questions did not specify nonmedical opioid use from medical opioid use, so it is possible that some estimates of prevalence (e.g., those using opioids) are inflated. While the study was open to both agricultural owners and workers, the quantitative study participants were predominantly owners, and all qualitative participants were owners. Therefore, the needs of agricultural workers could not be well-represented. There were not alternative versions of this survey for individuals who speak Spanish or other languages, and it is thus limited to an English-speaking population. Finally, it is notable that these data were collected both prior to and early on in the COVID-19 pandemic. There is evidence to suggest that those in agricultural industries have experienced significant increases in financial and psychological stressors related to the pandemic, which could further exacerbate the problems experienced by this population [21,22].

## 5. Conclusions

This study examined opioid use and its impact among agricultural industries (nursery/landscape and crops/livestock) using both quantitative and qualitative methods. Participants represented both business owners and workers. The results of this study suggest that the effects inflicted by the opioid epidemic on the agricultural community are potentially far-reaching. There is a need to conduct a greater-scale study to more fully characterize the effects of the opioid crisis across age and gender as well as industry with a larger sample. Follow-up qualitative interviews for richer data and information regarding the varied pathways into and out of opioid use will be beneficial moving forward. Lawmakers and community decision makers may consider allocating additional resources to prevention efforts and treatment availability, specifically for rural areas. Data from this study show the prevalence of opioid use and associated deleterious consequences in agricultural industries are high, but access to education and treatment to combat the opioid crisis may be relatively low. Additional research assessing individual access and perceptions of access to opioid treatment (e.g., pharmacotherapy, individual and group therapy) in agricultural industries would substantially add to this discussion. Finally, tailored interventions targeting injury prevention and pain management would be beneficial for agricultural populations and particularly for horticultural industries, which seem to have differentially higher pain and opioid use. Both owners and employees of agricultural industries appear to be at higher risk for opioid use and potential misuse than the general population, and further research and intervention support for this population is warranted.

## Figures and Tables

**Table 1:** Quantitative Participant Demographics.

Variable		Full Sample	Crops, Livestock, and Other	Nursery and Landscaping
		*n* = 129	*n* = 49	*n* = 80
Industry				
	Production (Crops, Livestock, and Other)	49 (38%)		
	Horticulture (Nursery and Landscaping)	80 (62%)		
Age Range				
	18–25	6 (4.7%)	1 (2%)	5 (6.2%)
	26–35	43 (33.3%)	17 (33.3%)	26 (32.5%)
	36–45	61 (47.3%)	24 (47.1%)	37 (46.2%)
	46–55	11 (8.5%)	4 (7.8%)	8 (10%)
	56–65	6 (4.7%)	3 (5.9%)	3 (3.8%)
	66–75	2 (1.6%)	2 (3.9%)	1 (1.2%)
Own/Co-Own Business				
	Yes	74 (57.4%)	21 (41.2%)	53 (66.2%)
	No	55 (42.6%)	30 (58.8%)	27 (33.8%)
Number Workers				
	0–20	19 (14.7%)	9 (18.4%)	10 (12.5%)
	21–40	39 (30.2%)	11 (22.4%)	28 (35%)
	41–60	16 (12.4%)	1 (2%)	15 (18.8%)

**Table 2:** Quantitative Survey Response Information.

Survey Question	Crops, Livestock, and Other	Nursery and Landscaping	*p*-Value
Proportion Workers Opioids			0.04
None of the workforce	6 (28.57%)	3 (5.66%)	
Less than half of the workforce	9 (42.86%)	36 (67.92%)	
About half of the workforce	5 (23.81%)	12 (22.64%)	
More than half of the workforce	1 (4.76%)	2 (3.77%)	
Turnover Increased			0.02
Yes	8 (38.1%)	33 (62.26%)	
No	7 (33.33%)	17 (32.08%)	
Not sure	6 (28.57%)	3 (5.66%)	
Business Impact			0.04
Decrease in productivity	3 (14.29%)	19 (35.85%)	
Decrease in employees	9 (42.86%)	18 (33.96%)	
Failed drug tests	1 (4.76%)	8 (15.09%)	
Employees come to work high	3 (14.29%)	6 (11.32%)	
Business has not been affected	4 (19.05%)	2 (3.77%)	
Other	1 (4.76%)	0 (0%)	
Familiar with Epidemic			0.003
Have never heard of it	3 (6.12%)	2 (2.5%)	
Have heard a few things but not much	9 (18.37%)	38 (47.5%)	
Have heard quite a few things, but it has not impacted me directly	23 (46.94%)	17 (21.25%)	
Have heard a lot about it, and it has had some impact on me	12 (24.49%)	18 (22.5%)	
Have heard a lot about it, and my life has been significantly impacted by it	2 (4.08%)	5 (6.25%)	
Personal Use			<0.001
Yes	12 (24.49%)	58 (72.5%)	
No	36 (73.47%)	22 (27.5%)	
Not sure	1 (2.04%)	0 (0%)	
Opioid Use Disorder Diagnosis			0.008
Yes	4 (30.77%)	42 (72.41%)	
No	9 (69.23%)	16 (27.59%)	
Experienced Pain			0.002
Yes	27 (55.1%)	64 (81.01%)	
No	22 (44.9%)	15 (18.99%)	
Work Injury Opioids			0.28
Yes	8 (61.54%)	46 (79.31%)	
No	5 (38.46%)	12 (20.69%)	
Work Loss Opioids			
Yes	3 (23.08%)	30 (51.72%)	0.07
No	10 (76.92%)	28 (48.28%)	
Know Users			<0.001
Yes	24 (48.98%)	65 (81.25%)	
No	25 (51.02%)	15 (18.75%)	

## Data Availability

Study approval and participant consent forms stated that study response information would not be shared outside of the research team unless in aggregate form. This was deemed prudent given the sensitive nature of the questions asked. Unfortunately, it is not possible to make participant data available to others at this time.
